# Effects of high-fat diet on thyroid autoimmunity in the female rat

**DOI:** 10.1186/s12902-022-01093-5

**Published:** 2022-07-16

**Authors:** Zhengzheng Liao, Ying Kong, Liang Zeng, Qing Wan, Jinfang Hu, Yaojun Cai

**Affiliations:** 1 Department of Pharmacy, the First Affiliated Hospital of Nanchang University, 330006 Nanchang, Jiangxi People’s Republic of China; 2 Department of Otorhinolaryngology, Head & Neck Surgery, the First Affiliated Hospital of Nanchang University, 330006 Nanchang, Jiangxi People’s Republic of China; 3 Department of Endocrinology and Metabolism, the First Affiliated Hospital of Nanchang University, 330006 Nanchang, Jiangxi People’s Republic of China; 4 Jiangxi Clinical Research Center for Endocrine and Metabolic Disease, Jiangxi 330006 Nanchang, People’s Republic of China; 5 Jiangxi Branch of National Clinical Research Center for Metabolic Disease, Jiangxi 330006 Nanchang, People’s Republic of China

**Keywords:** High-fat diet, Thyroid autoimmunity, PD-1, PD-L1, Lymphocytic infiltration

## Abstract

**Background:**

While contributions of dyslipidemia to autoimmune diseases have been described, its impact on thyroid autoimmunity (TA) is less clear. Programmed cell death 1(PD-1)/PD-ligand 1 (PD-L1) immune checkpoint is crucial in preventing autoimmune attack while its blockade exacerbates TA. We thus unveiled the effect of high-fat diet (HFD) on TA, focusing on the contribution of PD-1/PD-L1.

**Methods:**

Female Sprague Dawley (SD) rats were randomly fed with a regular diet or HFD (60% calories from fat) for 24 weeks. Then, thyroid ultrasonography was performed and samples were collected for lipid and thyroid-related parameter measure.

**Results:**

HFD rats exhibited hyperlipemia and abnormal biosynthesis of the unsaturated fatty acid in serum detected by lipidomics. These rats displayed a relatively lower echogenicity and increased inflammatory infiltration in thyroid accompanied by rising serum thyroid autoantibody levels and hypothyroidism, mimicking human Hashimoto’s thyroiditis. These alterations were concurrent with decreased mRNA and immunostaining of intrathyroidal PD-1 and also serum PD-1 levels but not the PD-L1 expression, suggesting a role of a PD-1 pathway. Meanwhile, the infiltration of B and T cell, a key cellular event inhibited by the PD-1 signals, was enhanced in the thyroid of HFD rats, along with thyroid fibrosis and apoptosis.

**Conclusions:**

Our data suggest that HFD triggers TA through a mechanism possibly involving downregulation of PD-1-related immunosuppression, providing a novel insight into the link between dyslipidemia and autoimmune toxicities.

**Supplementary Information:**

The online version contains supplementary material available at 10.1186/s12902-022-01093-5.

## Introduction

Autoimmune thyroiditis (AIT) is a frequent autoimmune disease susceptible to women [1]. Hashimoto’s thyroiditis (HT) represents the principal form of AIT leading to hypothyroidism. According to several epidemiological reports [2, 3], an increased incidence of HT have been described, although the reasons for such an increase remain unclear. HT is typically characterized by thyroid fibrosis associated with lymphocyte infiltration as well as the presence of autoantibodies against thyroid-peroxidase (TPOAb) and thyroglobulin (TgAb). The existence of such autoantibodies is the most prevalent autoimmune state in the general population with the prevalence around 14.19% (10.19% of positive TPOAb, 9.70% of positive TgAb) [3].

Dietary factors, especially excessive or deficient intake of iodine, have been reported to be implicated in the pathogenesis of adverse thyroid conditions, such as HT and hypothyroidism [4]. Interestingly, increasing evidence has revealed that excessive fat intake alters the thyroid hormone status. A population-based case-control study included 24,100 subjects has reported an association of dyslipidemia with approximately 35% increased risk for subclinical hypothyroidism [5]. More recently, a prospective cohort study has shown that higher serum lipid level was an independent risk factor for progression to overt hypothyroidism [6]. Using animal models, the study by Shao et al. has demonstrated that 24 weeks of high-fat diet (HFD) decreased serum T4 and T3 levels in parallel with elevated concentrations of thyrotropin (TSH), indicating hypothyroidism [7]. These observations imply that the thyroid gland is susceptible to aberration following an excessive fat intake. On the other hand, clinical data from recent years have shown that HT patients had abnormal serum lipid levels regardless of thyroid function [8–12] and high serum low-density lipoprotein levels were associated with an increased risk of positive thyroid autoantibodies [10]. Moreover, lipid-lowering agents have been shown to reduce thyroid antibody titers and improve thyroid function in HT patients [13–16]. Given these findings, it seems likely that excess lipid uptake may contribute to the pathogenesis of HT, but the exact relationship and molecular mechanism between excessive dietary fat and thyroid autoimmunity (TA) is less clear.

The programmed cell death 1(PD-1) / PD-ligand 1 (PD-L1) signaling is crucial in regulating immune tolerance, essentially placing a “brake” on the activation of the immune system [17]. PD-1 is mainly expressed on the surface of the T cells, B cells and myeloid cells, with its main ligand PD-L1, expressed on the antigen-presenting cells. Stimulation of PD-1 by its ligand inhibits the TCR signaling through the blocking of the PI3K/AKT pathway, resulting in the suppression of the effector T cell function and protecting target tissues from their attack [18]. Conversely, blockade of PD-1/PD-L1 leads to the activation of an autoimmune response against tissues, known as immune-related adverse events (irAEs). Indeed, with the increasing use of PD-1 or PD-L1 inhibitors in cancer immunotherapy, autoimmune thyroid diseases have been frequently described, with thyroiditis (38%) reported as the most common first irAEs in patients receiving anti-PD-L1 immunotherapy [19]. The study by Osorio et al. [20] has shown that thyroid autoantibodies were present in most patients developing anti-PD-1 thyroid dysfunction (80% versus 8%). Tg-induced thyroiditis is an ideal animal model for HT in humans [21, 22]. In animal studies, a decrease in the PD-1/PD-L1 pathway has been observed in the placenta and spleen of HT mice [23, 24] and blocking the PD-1/PD-L1 interaction with antibody exacerbated the development of thyroiditis [25, 26]. These observations strongly support the hypothesis of a unique role of PD-1/PD-L1 in the pathogenesis of HT. Therefore, in the current study, we aimed to unveil the effect and the underlying mechanism of excessive dietary fat on TA, especially the PD-1/PD-L1 signaling.

## Materials and methods

### Animals

Female SD rats (6–7 weeks old; 170 ~ 210 g) were obtained from Medical School of Nanchang University and kept in the controlled laboratory conditions (22 ± 2 °C, 55 ± 5% humidity, a 12-h light/12-h dark cycle) with free access to standard chow diet and water, prior to the dietary manipulation. After one-week of acclimatization, rats were randomly assigned to the HFD group (*n* = 10) fed with a high-fat diet (D12492, 60% fat, 20% carbohydrate, 20% protein; Research Diets, USA) and the control group (*n* = 10) fed with a chow diet (D12450B, 10% fat, 70% carbohydrate, 20% protein; Research Diets, USA), for a period of 24 weeks.

### Thyroid ultrasound

At week-24, an ultrasound scan of the thyroid gland was performed with a LOGIQ V5 Expert color doppler ultrasound (GE Medical Systems, Wuxi, China), using a 10.0-MHz transducer. The rats were secured on the heated platform stage with tape, hairs were removed from the neck to the high thorax area with depilatory cream and electric shaver after anesthetizing with 3% sodium pentobarbital (i.p.). All ultrasonographic assessments were conducted by the same trained sonographer blinded to the experimental groups.

### Tissue preparation

All the rats were fasted for 12 h and blood samples were randomly obtained from the suborbital vein, which then centrifuged for assessing serological parameters. Next, the rats were sacrificed after deep anesthetization, and the thyroid tissue samples were collected and randomly assigned for further assays: left thyroid gland tissues for H&E, Masson’s trichrome, TUNEL staining and immunohistochemistry and right thyroid gland tissues for real-time RT-PCR.

### Serological parameters

The kits for measuring fasting serum total cholesterol, triglycerides, and low-density lipoprotein cholesterol were obtained from the Institute of the Jiancheng Bioengineering (Nanjing, China). The serum levels of TSH, soluble PD-L1(sPD-L1), and soluble PD-1 (sPD-1) were analyzed utilizing enzyme linked immunosorbent assay (ELISA) kits (TSH and sPD-L1: USCN Life Science Inc., China; sPD-1: RayBiotech Co. Ltd., USA). The serum levels of triiodothyronine (T3), tetraiodothyroxine (T4), TPOAb, and TgAb were quantified using electrochemiluminescence immunoassay (ECLIA) on Cobas e411 immunoassay analyzer (Roche, Germany) as previously described [21, 22]. All assays were performed according to the manufacturers’ guidelines.

### Lipidomic analysis

Serum samples (*n* = 6) were analyzed by liquid chromatography-mass spectrometry (LC/MS) based on a Thermo Ultimate 3000 UPLC system coupled to a Q Exactive Focus mass spectrometer (Thermo Fisher Scientific, Germany). The measurements were conducted at BioNovoGene Technology Co., Ltd (Suzhou, China). The detailed methods were provided in the Additional file 1.

### Histopathological evaluation

For histopathology, the tissues from the left thyroid glands were fixed in paraffin and then cut into 5-µm noncontiguous Sect. (1/10 serial sections), and subjected to H&E, Masson’s trichrome, and TUNEL staining. All thyroid sections were examined microscopically for histological changes by two experienced observers blinded to the experimental protocol. Histological evaluation for thyroiditis was based on the extent of monocyte infiltration [21]: 0 = absence of infiltrate; 1 = interstitial accumulation of inflammatory cells around one or two follicles; 2 = one or two foci of inflammatory cells reaching the size of a follicle; 3 = 10–40% inflammatory cells infiltration; 4 = greater than 40% inflammatory cells infiltration. A score below 1 indicated non-significant pathology.

### Immunohistochemistry (IHC)

For IHC analysis, five noncontiguous Sect. (1/5 serial sections) per antibody were used. Thyroid sections were dewaxed, hydrated, and then antigen retrieval was performed. Endogenous peroxidase was quenched and sections were blocked in serum. Then, sections were incubated with the appropriate primary and secondary antibodies. The primary antibodies were PD-1 (86,163, Cell Signaling, 1:200), PD-L1 (13,684, Cell Signaling, 1:50), CD45 (13,917, Cell Signaling, 1:200), CD4 (25,229, Cell Signaling, 1:50), CD8 (85,336, Cell Signaling, 1:200), and CD19 (90,176, Cell Signaling, 1:800). Finally, chromogen was added to each slice and further stained with hematoxylin. IHC examinations were performed by two experienced observers blinded to the experimental protocol, using magnifications ranging from 400 to 2,000×.

### RNA purification and real-time RT-PCR

Total RNA was extracted from the thyroid gland using the TRI reagent (Invitrogen) and treated with RNase-free DNase, followed by reverse transcription with AMV (Promega) according to the manufacturer’s protocol. Sequences of the primers for PCR involved were as follows: *18 S*: Forward Primer (FP) - GTAACCCGTTGA ACCCCATT, Reverse Primer (RP) - CCATCCAATCGGTAGTAGCG; *PD-1*: FP - CAGCTTGTCC AACTGGTCG, RP - GCTCAAACCATTACAGAAGGCG; *PD-L1*: FP - GCTCCAA AGGACTTGTACGTG, RP - TGATCTGAAGGGCAGCATTTC. The relative thyroid gland mRNA expression was analyzed using the 2^−ΔΔCt^ method and normalized to the expression of *18s* rRNA.

### Statistical analysis

All data were expressed as the mean ± standard error of means (SEM). For normally distributed data, differences between the groups were analyzed by unpaired Student’s t test. For nonparametric data, differences between groups were analyzed by a Mann-Whitney U test. A *p* < 0.05 was considered statistically significant. Statistical analysis was performed using the Graphpad Prism 5.0 (San Diego, USA).

## Results

### Building a HFD model in female rats

**Fig. 1:**
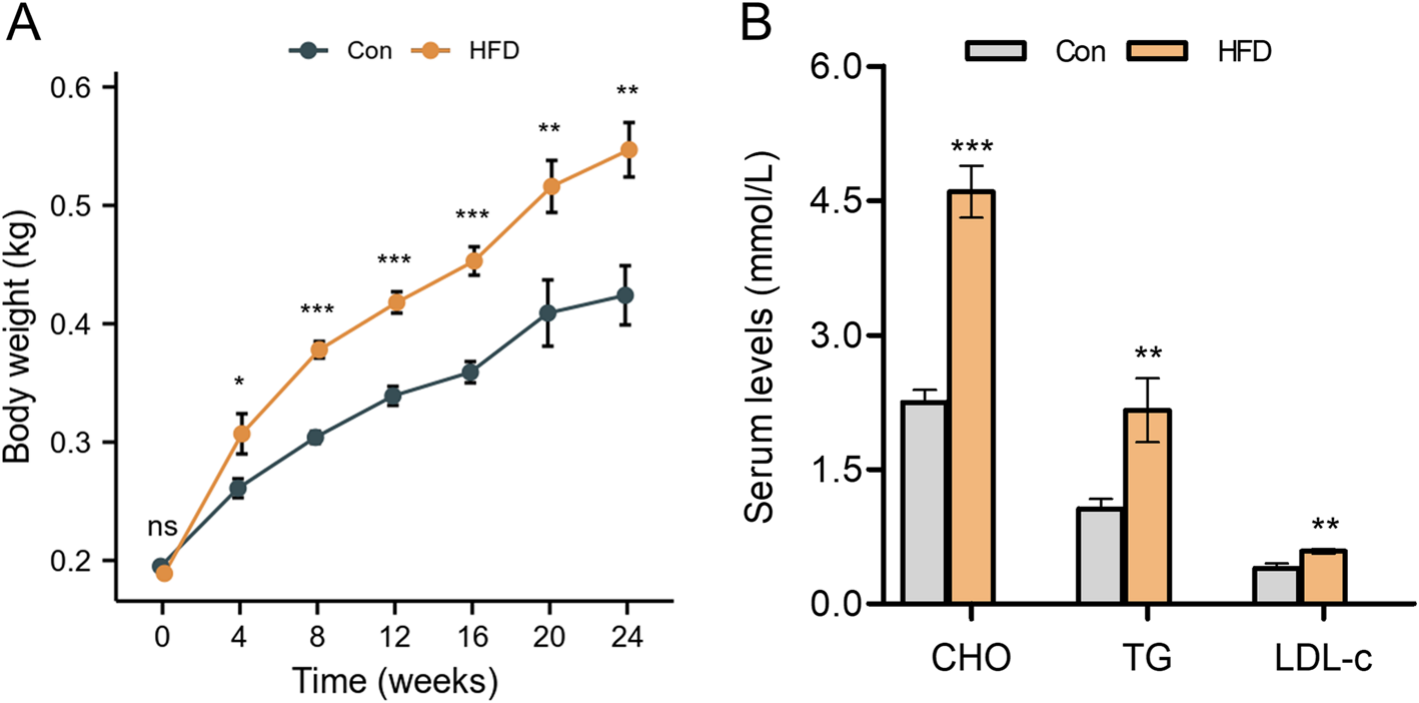
Building a HFD model in female rats. **A** Body weights. **B** Serum levels of CHO, TG, and LDL-c. Values were mean ± SEM, *n* = 10; ^*^p < 0.05, ^**^p < 0.01, and ^***^p < 0.001

The body weights of rats were monitored every four weeks. At baseline, all rats had similar body weights, but the rats in the HFD group gained significantly more weight than those in the control group during the 24-week feeding period (Fig. 1A). To assess the effects of HFD on serum lipid profiles, levels of total cholesterol (CHO), triglycerides (TG), and LDL-cholesterol (LDL-c) were measured. At week-24, the rats in the HFD group displayed a significant increase in serum levels of CHO, TG, and LDL-c than in the control group (Fig. 1B).

### HFD induced changes in serum lipidome

**Fig. 2:**
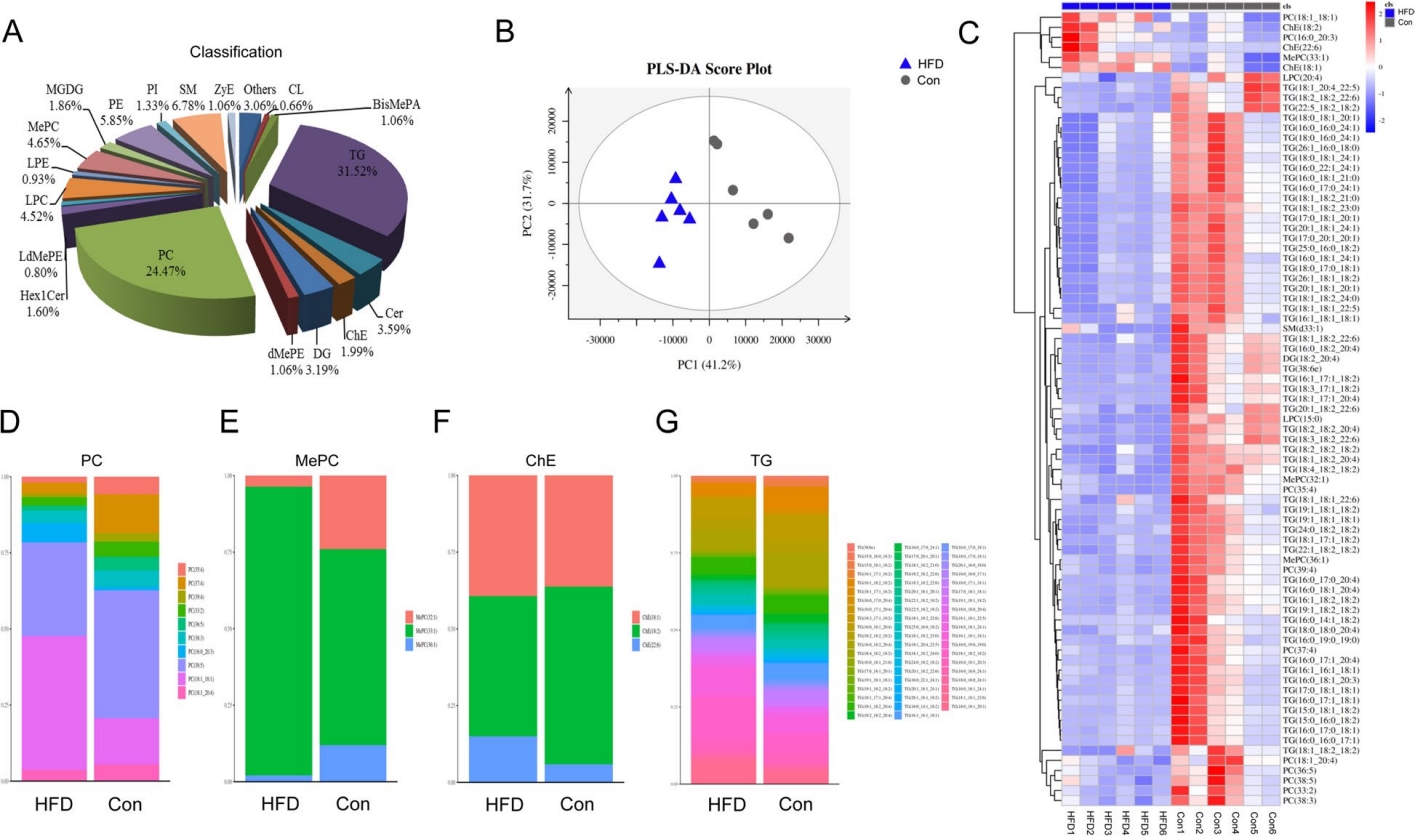
HFD induced changes in serum lipidome. **A** Classification of the serum lipid by the biological process. **B** The OPLS-DA score plot derived from the LC-MS of serum obtained from the HFD (blue) and control (grey) groups. **C** Heatmap presentation of differential lipid metabolites. The relative values were presented by color differences, with columns representing samples and rows representing lipids. **D**-**G** The content in four main kinds of differential lipids (PC, MePC, ChE, and TG) were statistically analyzed (*n* = 6)

Evidence has indicated that abnormal metabolism of lipid molecules is the main cause of cytotoxicity caused by HFD [27]. In this study, the LC-MS method was employed to compare the differences in serum lipid metabolite profiles between the two groups. A total of 752 individual lipid metabolites were identified in the light of the Lipid Map Database, and divided into 19 categories (Fig. 2A). The statistical evaluation by the partial least-square discriminant analysis (PLS-DA) model revealed a clear discrimination in two groups, characterized by R^2^Y = 98.4% and Q^2^ = 0.913 (Fig. 2B). Meanwhile, the orthogonal partial least-square discriminant analysis (OPLS-DA) also showed that the Con and HFD groups could be separated, in line with the PLS-DA result (see Additional file 2). Additionally, the fold-change in these lipid metabolites was processed by hierarchical clustering, and the relative upregulation (red) or downregulation (blue) of lipid level in the control and HFD groups were exhibited by a heatmap (Fig. 2C). The dysregulated lipid metabolites included 10 phosphatidyl-cholines (PCs), 3 methylphosphocholines (MePCs), 3 cholesteryl esters (ChEs), 59 triglycerides (TGs), 2 lysophosphatidylcholines (LPCs), 1 diglyceride (DG) and 1 sphingomyelin (SM) (Fig. 2D-G and Additional file 3).

### HFD triggered the development of HT-like autoimmunity

**Fig. 3:**
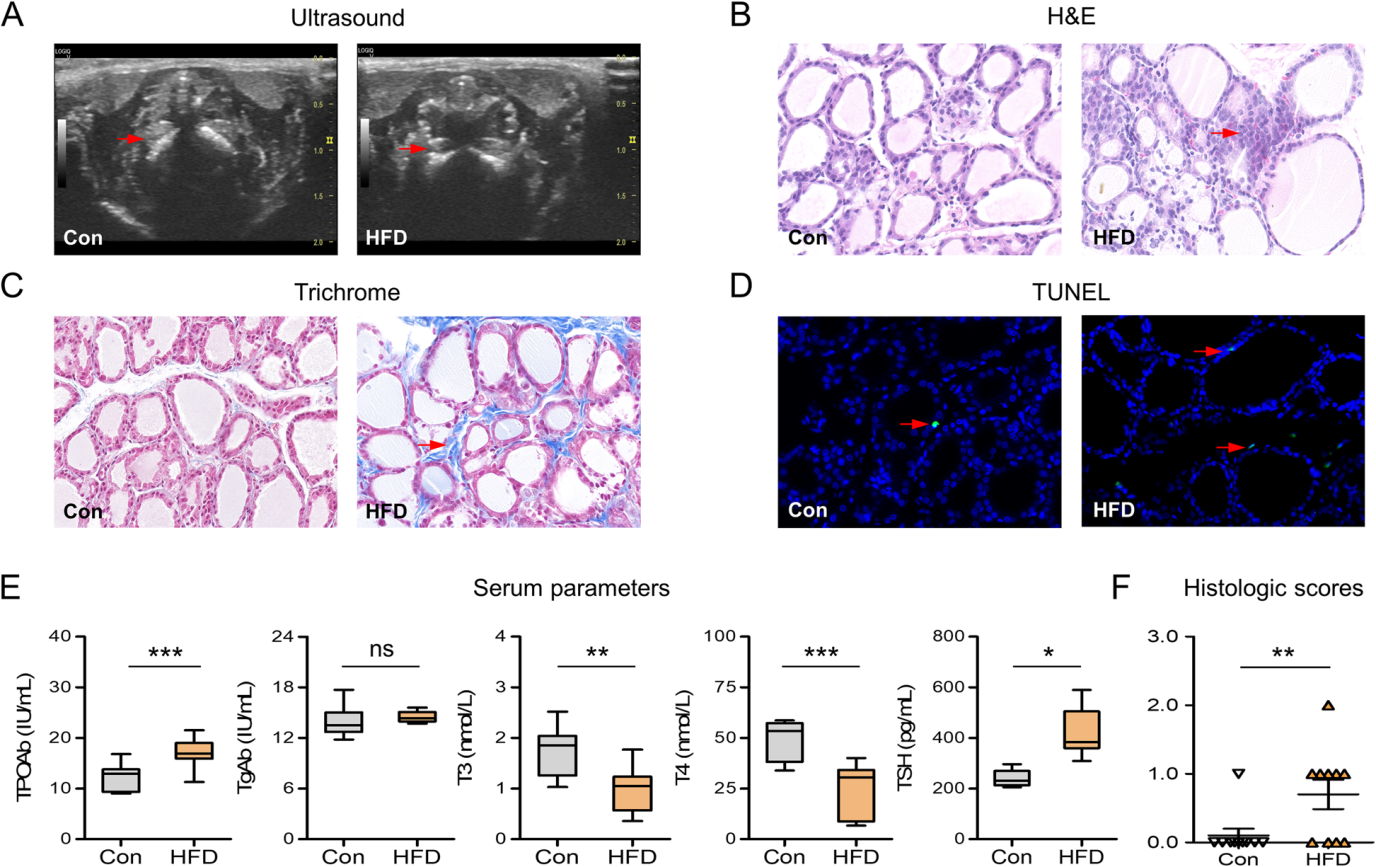
HFD triggered the development of HT-like autoimmunity. **A** Representative thyroid images under ultrasound scanning (*n* = 5). Red arrows: thyroid gland. **B** Representative thyroid sections stained with H&E (*n* = 10; Magnification ×400). Red arrows: infiltrated monocytes. **C** Representative thyroid sections stained with Masson’s trichrome (*n* = 5; Magnification ×400). Red arrows: thyroid fibrosis. **D** Representative immunofluorescence staining images showing TUNEL-labeled (green) apoptotic cells counterstained with DAPI (blue) in thyroids (*n* = 5; Magnification×400). Red arrows: apoptotic cells. **E** Serum levels of thyroiditis-related parameters, including TPOAb, TgAb, T3, T4 and TSH (*n* = 10). **F** Quantitation of the degree of monocyte infiltration in thyroids using H&E. Values were mean ± SEM; ^*^p < 0.05, ^**^p < 0.01, and ^***^p < 0.001; ns, no statistical significance

The echo structure of the thyroid parenchyma was detected by ultrasound scanning at week-24 (Fig. 3A). The thyroid glands of control rats exhibited a homogeneous appearance, while the HFD rats showed thyroids with relatively hypoechoic heterogeneous echotexture, consistent with a previous study [7]. Histological examination (Fig. 3B) revealed that the thyroids of control rats exhibited a normal feature with moderately sized follicles that were evenly distributed, and monocyte infiltration was hardly observed. In contrast, HFD-fed rats displayed disordered thyroid follicles with flat follicular epithelial cells, and monocyte infiltration was found more or less in thyroids. Further quantitative analysis (Fig. 3F) showed that the severity score of thyroiditis in the HFD group was significantly higher than that in controls. Furthermore, compared with the controls, there was significantly increased thyroid fibrosis (Fig. 3C) and apoptosis (Fig. 3D) in the HFD group. Besides, serum levels of TPOAb, a key biomarker of HT, were significantly increased in the HFD group (Fig. 3E), while corresponding decreased levels of T3 and T4 in parallel with elevated levels of TSH in serum were also observed.

### HFD disturbed the PD-1 signaling in thyroid and serum

**Fig. 4:**
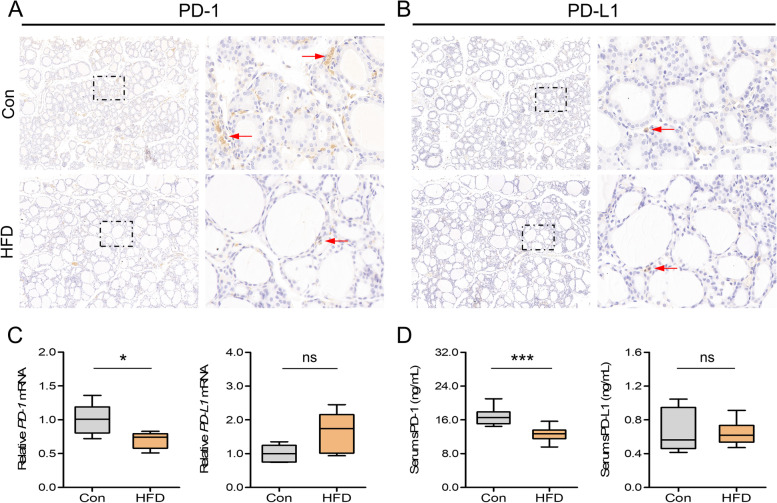
HFD disturbed the PD-1 signaling in thyroid and serum. **A**, **B** Representative images of PD-1 and PD-L1 staining in the thyroid (5 rats per group). Each right-hand panel (× 400) depicts a magnified image of the boxed area of the corresponding image in the left panel (× 100). **C** Relative mRNA expression of PD-1 and PD-L1 in the thyroid (*n* = 5). **D** Serum expression of sPD-1 and sPD-L1 (*n* = 10). Values were mean ± SEM; ^*^p < 0.05, and ^***^p < 0.001; ns, no statistical significance

IHC, real-time RT-PCR, and ELISA were performed to determine the expression levels of PD-1 and PD-L1 in the thyroid and serum (Fig. 4). There was an apparent decrease of PD-1 immunostaining in the HFD group compared to that in controls, while no difference in the PD-L1 expression was observed (Fig. 4A, B), which was confirmed by the quantitative analysis of the *PD-1* and *PD-L1* mRNA levels in the thyroid (Fig. 4C). Given that the PD-1/PD-L1 are detectable in both the membrane and soluble forms, we also determined the serum levels of sPD-1and sPD-L1 using ELISA, which revealed that sPD-1 levels were significantly lower in the HFD group than in controls (Fig. 4D). However, no significant difference in the expression level of sPD-L1 was observed between the two groups.

### HFD increased lymphocyte infiltration in the thyroid

**Fig. 5:**
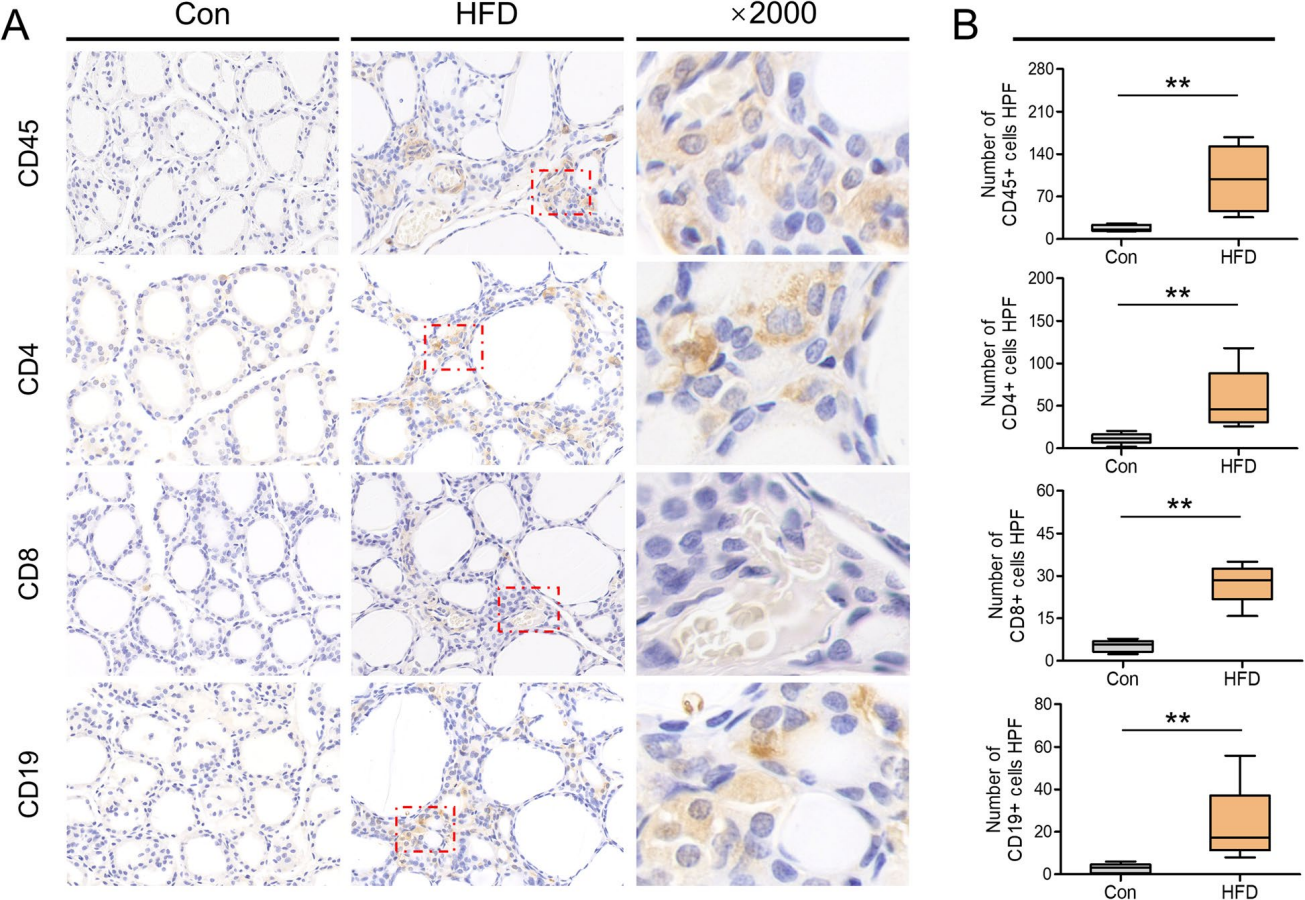
HFD increased lymphocyte infiltration in the thyroid. **A** Representative images of CD45 (mainly lymphocytic lineage), CD4 (CD4^+^ T cell), CD8 (CD8^+^ T cell) and CD19 (B cells) staining of thyroid sections. Each right panel (magnification ×2000) depicted a magnified image of the boxed area (red) of the corresponding image in the middle panel (magnification ×400). **B** Quantification of the data displayed in (**A**) revealed an increased infiltration of thyroid parenchyma by the CD45^+^ cells, CD4^+^ T cells, CD8^+^ T cells and B cells. Cells have been randomly counted in five nonoverlapping fields (magnification ×400) of tissue sections from 5 rats per group. Values were mean ± SEM; ^**^p < 0.01

Given that our data clearly demonstrated the insufficient PD-1 signaling stimulus in HFD rats, we explored further the key cellular events mediating by PD-1 inhibitory signals, such as lymphocyte infiltration [28, 29]. As depicted in Fig. 5A, IHC examination showed that in the thyroids of HFD rats, interfollicular spaces were filled with clusters of CD45 (lymphocytic lineage), CD4 (CD4 + T cells), CD8 (CD8 + T cells) and CD19 (B cells), which were mostly absent in the controls. Further quantitative analysis confirmed these results (Fig. 5B).

## Discussion

The adoption of poor dietary habits in the past few decades has resulted in a profound adverse impact on the nutritional status of both the disease-plagued and healthy populations [30]. Overnutrition has been associated with numerous risks in disease pathogenesis and progression [31]. In particular, a typical ‘Western diet’, that is rich in fat, protein or salt, has been related to immunosuppression, resulting in susceptibility to several types of autoimmune disorders [32, 33]. Although studies have shown that excessive fat intake interferes with thyroid function, it is not currently known whether it causes TA. HFD-fed rats are classical animal models used to investigate the adverse effects of fat intake in vivo [7, 34]. In this study, rats exposed to HFD exhibited a significant increase in serum CHO, TG and LDL-c levels relative to controls. Further lipidomic analysis revealed that HFD altered the biosynthesis of unsaturated fatty acids. For these reasons, our study used this model to investigate the direct effect of high-fat intake on TA and to shed light on the mechanism of these effects.

Indeed, a higher risk of autoimmune disorders has been previously demonstrated in the HFD animal models of systemic lupus erythematous [35], inflammatory bowel diseases [36], rheumatoid arthritis [37], multiple sclerosis [38], and autoimmune hepatitis [39]. Moreover, studies have shown that fat intake may affect autoantibody production and exacerbate the course of autoimmune diseases [40], suggesting a direct relationship between dyslipidemia and autoimmune toxicities. It is generally recognized that autoimmune conditions share similar characteristics (the autoimmune tautology), such as gender distribution, age of onset, genetic factors, and physiopathological mechanisms [41]. In this study, the ultrasound imaging revealed that HFD rats exhibited relatively hypoechoic heterogeneous echotexture in thyroids, as was observed in HT [1]. Further histopathological evaluation confirmed a significantly higher histological score for monocyte infiltration, along with thyroid fibrosis and apoptosis. More importantly, these observations were accompanied by rising serum TPOAb levels as well as hypothyroidism. These results demonstrated consumption of high-fat triggered thyroiditis with histological, clinical and serological signs mimicking human HT, consistent with a very recent clinic study reporting a positive correlation between increased consumption of animal proteins, saturated fats, and thyroid autoantibodies in HT patients [42]. These findings not only encourage future studies to explore the potential intervention with dietary fat restriction in the management of TA, but also to uncover the potential mechanisms implicating a HFD with TA.

Thyroid-peroxidase (TPO) is located in the thyroid follicular epithelial and its autoantibody, TPOAb, is a key serological biomarker of HT, which mediates the thyroid damage via autoimmune process [43]. Indeed, we observed an increased thyroid fibrosis and apoptosis concurrent with rising TPOAb levels among the HFD rats. A previous study has highlighted that serum TSH can enhance the expression/activity of TPO and then stimulates the synthesis of corresponding antibody [44], suggesting that the TPOAb increment can be partly explained by the elevated serum TSH. However, in vivo and in vitro studies have demonstrated that high-fat stimulation downregulates mRNA expression and activity of TPO in thyrocytes [45, 46]. These indicate the presence of other molecular mechanisms mediating the effect of HFD on TPOAb increment.

The success of the PD-1/PD-L1 blockade in cancer immunotherapy has attracted great attention to the putative role of PD-1/PD-L1 in the development of pathogenic autoimmunity. PD-1 maintains self-tolerance upon binding to its ligands, while the loss of PD-1 gene leads to autoimmunity diseases, such as type 1 diabetes, autoimmune myocarditis and lupus [47]. Studies have shown that blocking PD-1/PD-L1 signaling with antibody increases the accumulation of antigen-specific lymphocytes within the target tissue, resulting in disease severity [48–50]. Conversely, sustained activation of the PD-1/PD-L1 pathway decreases severity of autoimmune disorders [51–54]. In the field of thyroid disease, emerging studies suggested that blocking the PD-1/PD-L1 interaction with antibody exacerbated the development of autoimmune thyroiditis [25, 26]. These evidences have clearly elucidated the role of PD-1/PD-L1 preventing autoimmune attack while its blockade exacerbates TA. In this study, our results showed a significant decrease in immunostaining and mRNA expression of PD-1 but not PD-L1 in the thyroids of HFD rats. Thus, we inferred that HFD-induced TA might be, at least partially, the reflection of down-regulation of PD-1 signaling.

Given that the co-stimulatory molecules are detectable in both the membrane and soluble forms, we determined the serum sPD-1and sPD-L1 levels and discovered a significant decrease in sPD-1 levels in the HFD group relative to controls, while no difference in sPD-L1 levels. sPD-1 is produced through proteolytic cleavage of membrane-bound forms or encoded by alternatively spliced *PD-1* mRNA [55].The soluble protein participates in the blood system and mediates certain immune functions. Several autoimmune disorders, such as rheumatoid arthritis [56], immune thrombocytopenia [57], systemic juvenile idiopathic arthritis [58] and pemphigus vulgaris [59] have been implicated with a defect in the PD1 pathway, whereby lower serum sPD1 levels were correlated negatively with the severity of the disease. This could be due to sPD-1 exhibiting ‘agonism’ with its functions similar to the membrane form in the regulation of immunity by inhibiting T-cell responses [59, 60]. From this point, our observation of a decrease in sPD-1 levels supported further that HFD induced a dysfunctional inhibitory capacity of PD-1, although the mechanisms involved need further investigation. A previous study have revealed that lipids have polar head groups and nonpolar side-chains, both of which can interact with proteins affecting protein structure and function [61]. Further studies are needed to determine the specific effect of the individual fatty acid interacting with PD-1/PD-L1 axis on immune regulation of TA.

Various cellular and molecular pathways mediating PD-1-related immunosuppression have been described. These pathways work together to prevent autoimmunity and maintain immune tolerance. Among these, inhibition of function and proliferation of T and B cells has been identified as the most critical mechanism [28, 29]. In our work, the observation of a significant decrement in PD-1 signals had promoted us to speculate that the absence of negative regulation of intrathyroidal PD-1 might lead to activation of self-reactive T and B cells, as is the case of HT. To test this hypothesis, we examined the extent of lymphocyte infiltration in the thyroids. In rats exposed to HFD, intrathyroidal lymphocytic cell infiltration was significantly enhanced, and at least consisted of clusters of CD4+, CD8 + and CD19 + lymphocytes, similar to the types of infiltrated inflammatory cells in experimental autoimmune thyroiditis [62]. These findings suggested that downregulating PD-1 in response to HFD might lead to the activation of intrathyroidal T and B cells, which could aggravate the thyroiditis [63].

There were several limitations to our study. Firstly, animals included in this study were females because studies have indicated that HT was more prevalent in females and with a female-to-male ratio of nearly 10:1 [1], and that the association between increasing serum lipid levels and thyroid antibody positivity become more pronounced in female subjects [64]. It was unclear whether HFD would have similar impacts on males. Secondly, we did not perform an analysis of PD-1 and PD-L1 expression in the peripheral blood mononuclear cells, which might provide additional information in characterizing the role of the PD-1/PD-L1 axis following HFD. Lastly, this study did not clarify the detailed mechanism through which HFD feeding downregulated PD-1 signals in rats.

## Conclusions

In summary, emerging studies have reported that dyslipidemia may render individuals vulnerable to immune abnormalities. Using an animal model our study provides further evidence for such arguments, showing for the first time that HFD rats developed thyroiditis with sonographic, histological and serological signs resembling human HT, with a mechanism possibly involving downregulation of PD-1-related immunosuppression. These findings provide a novel insight into the link between dyslipidemia and autoimmune toxicities and offer preliminary evidence that making certain metabolism-related changes, such as dietary fat restriction, may contribute to the prevention of thyroid autoimmunity.

## Supplementary Information

**Additional file 1.** Supplementary Methods.

**Additional file 2.** The OPLS-DA score plot derived from the LC-MS of serum obtained from theHFD (blue) and control (grey) groups.

**Additional file 3:** **Supplemental Table 1. **The sub-lipid contentin differential lipids between the two groups ($\overline{\mathrm x}$ ± SEM, *n* = 6 per group).

## Data Availability

Data and material would be supplied based on reasonable request. If someone wants to request the data, please email ndyfy06200@ncu.edu.cn.

## Abbreviations

TA: Thyroid autoimmunity
HFD: High-fat diet
AT: Autoimmune thyroiditis
HT: Hashimoto’s thyroiditis
TPOAb: Thyroid-peroxidase antibody
TgAb: Thyroglobulin antibody
T3: Triiodothyronine
T4: Tetraiodothyroxine
TSH: Thyrotropin
PD-1: Programmed cell death 1
PD-L1: Programmed cell death ligand 1
irAEs: Immune-related adverse events
ECLIA: Electrochemiluminescence immunoassay
ELISA: Enzyme-linked immunosorbent assay
LC/MS: Liquid chromatography-mass spectrometry
IHC: Immunohistochemistry
CHO: Cholesterol
TG: Triglycerides
LDL-c: LDL-cholesterol
PLS-DA: Partial least-square discriminant analysis
PCs: Phosphatidyl-cholines
MePCs: Methylphosphocholines
ChEs: Cholesteryl esters
LPCs: Lysophosphatidylcholines
DG: Diglyceride
SM: Sphingomyelin
