# Supplementary material for: Effects of high-fat diet on thyroid autoimmunity in the female rat
Source: BMC Endocr Disord. 2022 Jul 16;22:179. doi: 10.1186/s12902-022-01093-5 (PMC9287994; doi:10.1186/s12902-022-01093-5)
Supplement: Supplementary file 1 — Additional file 1. Supplementary Methods. [file 12902_2022_1093_MOESM1_ESM.docx]

**Additional file 1. Supplementary Methods.**

**Liquid chromatography-mass spectrometry (LC/MS)**

Transfer 100 μL of each serum sample into 2 mL centrifuge tubes and added 750 μL of Chloroform methanol mixed solution (2:1) (pre-cooled at -20 ℃), vortex for 30 s and then put on the ice for 40 min, add 190 μL H_2_O, vortex for 30 s, and still put on the ice for 10 min. Next, the sample was centrifuged at 12000 rpm for 5 min at room temperature and transfer 300 μL lower layer fluid into a new centrifuge tube and added 500 μL of Chloroform methanol mixed solution (2:1) (pre-cooled at -20 ℃), vortex for 30 s. Then, the blood samples were centrifuged at 12000 rpm for 5 min at room temperature and transfer 400 μL lower layer fluid into the same centrifuge tube above. Samples were concentrated to dry in vacuum and next the dissolve samples with 200 μL isopropanol, and the supernatant was filtered through 0.22 µm membrane to obtain the prepared samples for LC-MS.

The chromatographic separation was accomplished in an Thermo Ultimate 3000 system equipped with an ACQUITY UPLC® BEH C18 (100 × 2.1 mm, 1.7 µm, Waters) column maintained at 50 ℃. The temperature of the autosampler was 8 ℃. Gradient elution of analytes was carried out with acetonitrile: water = 60:40 (0.1% formic acid +10 mM ammonium formate) (C) and isopropanol: acetonitrile = 90:10 (0.1% formic acid +10 mM ammonium formate) (D) at a flow rate of 0.25 mL/min. Injection of 2 μL of each sample was done after equilibration. An increasing linear gradient of solvent C (v/v) was used as follows: 0~5 min, 70~57% C; 5~5.1 min, 57%~50% C; 5.1~14 min, 50%~30% C; 14~14.1 min, 30% C; 14.1~21 min, 30%~1% C; 21~24 min, 1% C; 24~24.1 min, 1%~70% C; 24.1~28 min, 70% C.

The ESI-MS^n^ experiments were executed on the Thermo Q Exactive Focus mass spectrometer with the spray voltage of 3.5 kV and -2.5 kV in positive and negative modes, respectively. Sheath gas and auxiliary gas were set at 30 and 10 arbitrary units, respectively. The capillary temperature was 325 ℃. respectively. The Orbitrap analyzer scanned over a mass range of m/z 150-2 000 for full scan at a mass resolution of 35000. Data dependent acquisition (DDA) MS/MS experiments were performed with HCD scan. The normalized collision energy was 30 eV. Dynamic exclusion was implemented to remove some unnecessary information in MS/MS spectra.
