# Supplementary material for: Effects of high-fat diet on thyroid autoimmunity in the female rat
Source: BMC Endocr Disord. 2022 Jul 16;22:179. doi: 10.1186/s12902-022-01093-5 (PMC9287994; doi:10.1186/s12902-022-01093-5)

**Additional file 2.** The OPLS-DA score plot derived from the LC-MS of serum obtained from the HFD (blue) and control (grey) groups.


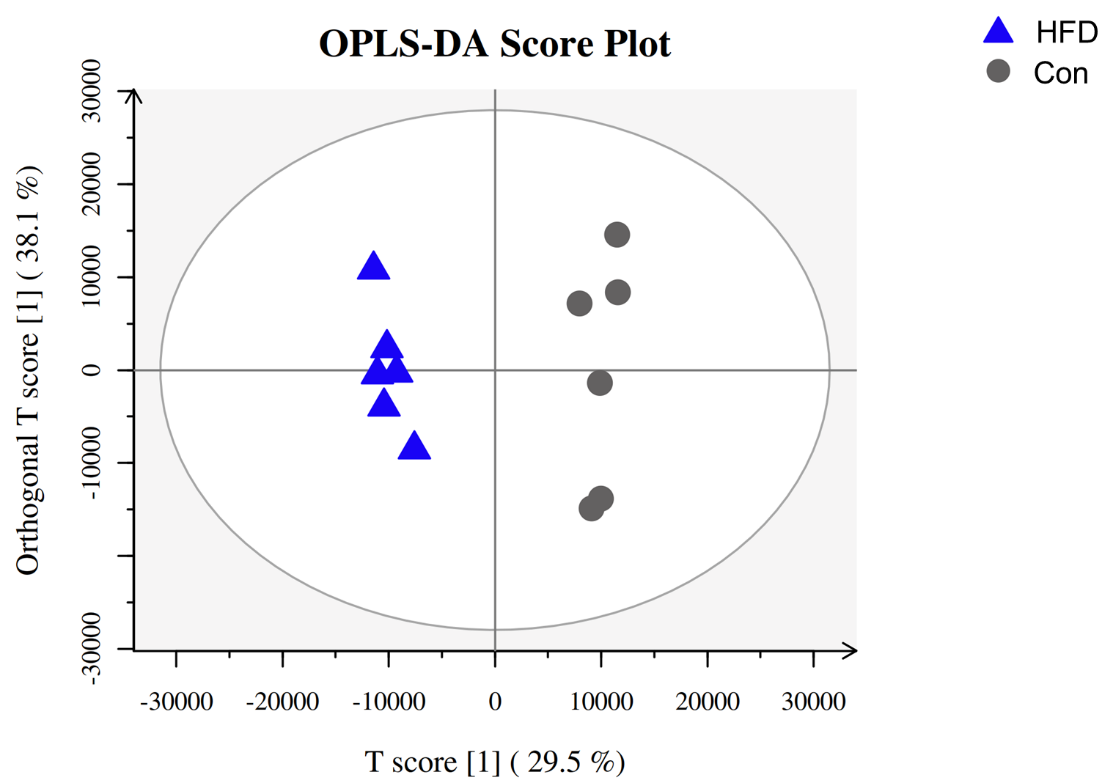

Supplement: Supplementary file 2 — Additional file 2. The OPLS-DA score plot derived from the LC-MS of serum obtained from theHFD (blue) and control (grey) groups. [file 12902_2022_1093_MOESM2_ESM.docx]
