# Supplementary material for: Effects of high-fat diet on thyroid autoimmunity in the female rat
Source: BMC Endocr Disord. 2022 Jul 16;22:179. doi: 10.1186/s12902-022-01093-5 (PMC9287994; doi:10.1186/s12902-022-01093-5)
Supplement: Supplementary file 3 — Additional file 3: Supplemental Table 1. The sub-lipid contentin differential lipids between the two groups ($\overline{\mathrm x}$x¯ ± SEM, n = 6 per group). [file 12902_2022_1093_MOESM3_ESM.docx]

**Additional file 3**

**Supplemental Table 1** The sub-lipid content in differential lipids between the two groups (x ± SEM, n = 6 per group)

| **Name** | **HFD** | **Con** | ***P* value** |
| --- | --- | --- | --- |
| PC (33:2) | 750409.8 ± 251393.5 | 2230469 ± 1158564 | *p*＜0.01 |
| PC (35:4) | 528023.9 ± 319958.5 | 2615536 ± 914645.8 | *p*＜0.01 |
| PC (37:4) | 1021029 ± 544466.3 | 5716081 ± 3608540 | *p*＜0.01 |
| PC (39:4) | 299438.4 ± 124489.1 | 1260172 ± 541896.6 | *p*＜0.05 |
| PC (36:5) | 457904 ± 372175.8 | 2004706 ± 1228482 | *p*＜0.05 |
| PC (38:3) | 1115649 ± 368605.2 | 2437662 ± 971049.4 | *p*＜0.01 |
| PC (38:5) | 8398379 ± 4587084 | 18837478 ± 8283290 | *p*＜0.05 |
| PC (16:0_20:3) | 1732055 ± 1211100 | 506094.3 ± 413538.6 | *p*＜0.05 |
| PC (18:1_18:1) | 12034627 ± 4208983 | 6864479 ± 2518432 | *p*＜0.05 |
| PC (18:1_30:4) | 985188.5 ± 552433.9 | 2418863 ± 1076709 | *p*＜0.05 |
| MePC (32:1) | 527482.1 ± 320783.1 | 2597912 ± 940077 | *p*＜0.01 |
| MePC (33:1) | 13602776 ± 2531177 | 6916212 ± 2710486 | *p*＜0.01 |
| MePC (36:1) | 298498.8 ± 118338.9 | 1286119 ± 579080.8 | *p*＜0.01 |
| ChE (18:1) | 2960856 ± 536181.1 | 1025870 ± 706168.4 | *p*＜0.05 |
| ChE (18:2) | 3436418 ± 1510656 | 1635573 ± 704697.1 | *p*＜0.05 |
| ChE (22:6) | 1128839 ± 1314958 | 165120.1 ± 81289.67 | *p*＜0.05 |
| TG (15:0_16:0_18:2) | 249366.7 ± 100960.1 | 1664534 ± 1321979 | *p*＜0.01 |
| TG (38:6e) | 53137.11 ± 17390.46 | 423802.9 ± 176838 | *p*＜0.01 |
| TG (15:0_18:1_18:2) | 533451.5 ± 222668.9 | 3421850 ± 2351957 | *p*＜0.01 |
| TG (16:1_17:1_18:2) | 141564.2 ± 81238.61 | 1155634 ± 614324.1 | *p*＜0.01 |
| TG (16:1_18:2_18:2) | 1503854 ± 739172.9 | 12397545 ± 6149935 | *p*＜0.01 |
| TG (18:1_17:1_18:2) | 291430 ± 156553.1 | 1629349 ± 822697.9 | *p*＜0.01 |
| TG (16:0_17:0_20:4) | 183118.5 ± 64679.81 | 1316996 ± 836571 | *p*＜0.01 |
| TG (16:0_17:1_20:4) | 118467.9 ± 54655.61 | 887007.4 ± 612547.4 | *p*＜0.01 |
| TG (18:3_17:1_18:2) | 24311.62 ± 5083.054 | 511995.4 ± 267155.2 | *p*＜0.01 |
| TG (16:0_18:1_20:4) | 2884135 ± 848774.1 | 14985883 ± 7632193 | *p*＜0.01 |
| TG (18:2_18:2_18:2) | 1656774 ± 1237373 | 6539218 ± 1716094 | *p*＜0.01 |
| TG (16:0_18:2_20:4) | 2737913 ± 1028293 | 20418972 ± 7336438 | *p*＜0.01 |
| TG (18:4_18:2_18:2) | 175258.8 ± 67615.1 | 532941.3 ± 123827.5 | *p*＜0.01 |
| TG (16:0_18:1_21:0) | 157517.6 ± 114361.4 | 652698.1 ± 237529.1 | *p*＜0.01 |
| TG (17:0_18:1_20:1) | 251250.5 ± 139461.3 | 1040572 ± 395459.4 | *p*＜0.01 |
| TG (19:1_18:1_18:1) | 210794.3 ± 101988.9 | 1136929 ± 492997.1 | *p*＜0.01 |
| TG (19:1_18:2_18:2) | 156057 ± 95217.63 | 1059439 ± 555326.8 | *p*＜0.01 |
| TG (18:1_17:1_20:4) | 78405.61 ± 36722.73 | 907805.4 ± 520397.4 | *p*＜0.01 |
| TG (18:1_18:2_20:4) | 2456903 ± 987703.7 | 10614548 ± 2150213 | *p*＜0.01 |
| TG (18:2_18:2_20:4) | 640662.4 ± 243837.2 | 4947332 ± 1150351 | *p*＜0.01 |
| TG (16:0_17:0_24:1) | 113070.4 ± 83605.07 | 508193.2 ± 174859 | *p*＜0.01 |
| TG (17:0_20:1_20:1) | 133770 ± 93610.9 | 693496.8 ± 240540 | *p*＜0.01 |
| TG (18:1_18:2_21:0) | 94885.05 ± 61214.35 | 594392.2 ± 214367.7 | *p*＜0.01 |
| TG (18:2_18:2_22:6) | 623084.2 ± 227506.4 | 3691679 ± 1556746 | *p*＜0.01 |
| TG (18:3_18:2_22:6) | 79791.09 ± 39744.46 | 555217.4 ± 154469.2 | *p*＜0.01 |
| TG (20:1_18:1_20:1) | 637047.5 ± 430638.9 | 2984641 ± 1320198 | *p*＜0.01 |
| TG (22:1_18:2_18:2) | 333989.4 ± 162808 | 1427060 ± 521628.6 | *p*＜0.01 |
| TG (22:5_18:2_18:2) | 124071.6 ± 64339.61 | 827230.4 ± 333487.1 | *p*＜0.01 |
| TG (18:1_18:2_22:6) | 1278569 ± 666232.7 | 4183008 ± 1438889 | *p*＜0.01 |
| TG (25:0_16:0_18:2) | 82307.82 ± 60462.46 | 481537.8 ± 166289.7 | *p*＜0.01 |
| TG (18:1_18:2_23:0) | 64649.05 ± 42447.87 | 457095.2 ± 156606.7 | *p*＜0.01 |
| TG (18:1_20:4_22:5) | 80528.82 ± 51139.63 | 486470.3 ± 255248.7 | *p*＜0.01 |
| TG (18:1_18:2_24:0) | 371836.7 ± 276328.5 | 1956517 ± 863760.8 | *p*＜0.01 |
| TG (24:0_18:2_18:2) | 258557 ± 171018.1 | 1460294 ± 694062.7 | *p*＜0.01 |
| TG (20:1_18:2_22:6) | 192760.3 ± 61449.46 | 538303.4 ± 183810.7 | *p*＜0.01 |
| TG (16:0_22:1_24:1) | 136487.6 ± 111676 | 658147.9 ± 263166.3 | *p*＜0.01 |
| TG (20:1_18:1_24:1) | 153650.4 ± 113894.4 | 930087.8 ± 388382.6 | *p*＜0.01 |
| TG (26:1_18:1_18:2) | 110030.4 ± 74154.2 | 805039.6 ± 370698 | *p*＜0.01 |
| TG (16:0_14:1_18:2) | 66176.01 ± 41567.84 | 976429.6 ± 1065978 | *p*＜0.01 |
| TG (16:1_16:1_18:1) | 1971201 ± 835756.5 | 9950590 ± 6996480 | *p*＜0.01 |
| TG (16:0_17:0_18:1) | 543310.7 ± 222913.3 | 2456230 ± 1806166 | *p*＜0.01 |
| TG (18:0_17:0_18:1) | 325246.8 ± 193682.9 | 1102558 ± 448246.1 | *p*＜0.01 |
| TG (26:1_16:0_18:0) | 202215.9 ± 166215.9 | 716284.7 ± 278618.3 | *p*＜0.01 |
| TG (16:0_16:0_17:1) | 388129.4 ± 119327.7 | 1835431 ± 1373510 | *p*＜0.01 |
| TG (16:0_17:1_18:1) | 1218344 ± 348681.8 | 6097862 ± 4892726 | *p*＜0.05 |
| TG (17:0_18:1_18:1) | 702872.2 ± 230425.6 | 2922515 ± 1834038 | *p*＜0.05 |
| TG (19:1_18:1_18:2) | 202773.6 ± 110664.3 | 851585 ± 478495.5 | *p*＜0.05 |
| TG (18:0_18:0_20:4) | 312785.1 ± 117566.9 | 871450.4± 465797.9 | *p*＜0.05 |
| TG (18:1_18:1_22:5) | 1030030 ± 512099.5 | 2629376 ± 795033.2 | *p*＜0.05 |
| TG (18:0_18:1_24:1) | 362312.9 ± 289146.9 | 1501118 ± 659082 | *p*＜0.05 |
| TG (16:1_18:1_18:1) | 3986979 ± 2100203 | 9961253 ± 4446976 | *p*＜0.05 |
| TG (16:0_19:0_19:0) | 368813.7 ± 114211.3 | 1219034 ± 761893 | *p*＜0.05 |
| TG (16:0_18:2_18:2) | 5876177 ± 3630297 | 11172220 ± 3906916 | *p*＜0.05 |
| TG (16:0_18:1_20:3) | 1610229 ± 334302.6 | 5658269 ± 3532370 | *p*＜0.05 |
| TG (16:0_16:0_24:1) | 571517 ± 448010.4 | 1767115 ± 747666.4 | *p*＜0.05 |
| TG (18:0_16:0_24:1) | 418489.5 ± 334798.4 | 1329839 ± 575677.1 | *p*＜0.05 |
| TG (16:0_18:1_24:1) | 565824.7 ± 437660.2 | 2371402 ± 1234978 | *p*＜0.05 |
| TG (18:1_18:1_22:6) | 1782595 ± 889215.1 | 3468270 ± 1521212 | *p*＜0.05 |
| TG (18:0_18:1_20:1) | 1254963 ± 847883.8 | 3391593 ± 1457095 | *p*＜0.05 |
| LPC (15:0) | 455295.2 ± 128746.5 | 2037821 ± 397210.4 | *p*＜0.01 |
| LPC (20:4) | 551506 ± 103430.6 | 943335.1 ± 191119.9 | *p*＜0.01 |
| DG (18:2_20:4) | 53137.11 ± 17390.46 | 423802.9 ± 176838 | *p*＜0.01 |
| SM (d33:1) | 741983.8 ± 350479.4 | 1424462 ± 464002.5 | *p*＜0.05 |
